# Does Obesity Affect the Rate of Force Development in Plantar Flexor Muscles among Older Adults?

**DOI:** 10.3390/sports12040089

**Published:** 2024-03-25

**Authors:** Hamza Ferhi, Elmoetez Magtouf, Ahmed Attia, Sylvain Durand, Sébastien Boyas, Bruno Beaune, Sabri Gaied Chortane, Wael Maktouf

**Affiliations:** 1 Research Laboratory (LR23JS01) Sport Performance, Health & Society, Higher Institute of Sport and Physical Education of Ksar Saîd, University of La Manouba, Tunis 2010, Tunisia; hamza.ferhi.etu@univ-lemans.fr (H.F.); elmoetez_bellah.magtouf.etu@univ-lemans.fr (E.M.); ahmedattias@gmail.com (A.A.); sabrigaied1@gmail.com (S.G.C.); 2 Laboratory Movement, Interactions, Performance (UR 4334), Department of Sport Sciences, Faculty of Sciences and Technologies, Le Mans University, 72000 Le Mans, France; sylvain.durand@univ-lemans.fr (S.D.); sebastien.boyas@univ-lemans.fr (S.B.); bruno.beaune@univ-lemans.fr (B.B.); 3 Bioengineering, Tissues and Neuroplasticity, UR 7377, Faculty of Health/EPISEN, University of Paris-Est Créteil, 8 rue du Général Sarrail, 94010 Créteil, France

**Keywords:** overweight, explosive force, triceps surae, walking

## Abstract

The literature offers limited information on the effect of obesity on the rate of force development (RFD), a critical parameter for mobility in older adults. The objectives of this study were to explore the influence of obesity on the RFD in older adults and to examine the association between this neuromuscular parameter and walking speed. The participants (42 older adults) were classified into two groups: the control group (CG, n = 22; mean age = 81.13 ± 4.02 years; body mass index (BMI) = 25.13 ± 3.35 kg/m^2^), and the obese group (OG, n = 20; mean age = 77.71 ± 2.95 years; BMI = 34.46 ± 3.25 kg/m^2^). Walking speed (m/s) was measured using the 10 m walking test. Neuromuscular parameters of the plantar flexors were evaluated during a maximal voluntary contraction test using a dynamometer. The RFD was calculated from the linear slop of the force–time curve in the following two phases: from the onset of the contraction to 50 ms (RFD_0–50_) and from 100 to 200 ms (RFD_100–200_). The gait speed was lower in the OG compared to the CG (*p* < 0.001). The RFD_50/100_ and RFD_100/200_ were lower in the OG compared to the CG (*p* < 0.001). The RFD_50/100_ was found to be the predominant influencer on gait speed in the OG. In conclusion, obesity negatively impacts the RFD in older adults and RFD stands out as the primary factor among the studied parameters influencing gait speed.

## 1. Introduction

The rate of force development (RFD) is a pivotal determinant of mobility in older adults, potentially having a greater influence than other muscular parameters [1]. The RFD parameter focuses on the force produced during the crucial initial 200 ms when a muscle is activated [2]. Intriguingly, this parameter exhibits heightened sensitivity to the aging process compared to maximal strength, underscoring its critical role in assessing age-related declines in muscle function [3]. Recent research has highlighted the RFD as a robust predictor of functional capabilities in older adults, notably due to its strong association with walking speed [1,4]. Furthermore, a diminished ability to generate swift force within the first 100–200 ms after a misstep might be a key factor in the diminished capacity of older adults to counteract falls [3]. Evaluations of RFD typically encompass various muscle contraction intervals, specifically the early (0–50 ms) and the latter (100–200 ms) phases [5]. The preliminary phase correlates with the onset of motor unit activation and its respective firing sequences, as well as intrinsic muscle characteristics like fiber composition and calcium dynamics [6]. Conversely, the latter phase largely draws influence from elements such as peak strength and overall muscular structure [7].

Prior studies have revealed age-related declines in maximal strength in lower limb muscles, including leg flexors [5], dorsiflexors [6], and plantar flexors (PF) [8]. Interestingly, it has been observed that the reduction in RFD during aging can be more pronounced (39–64%) than the decrease in maximal isometric strength (29–46%) [3,5]. In older adults with obesity, the accumulation of fat within skeletal muscles reduces the contractile component of the overall muscle volume [9]. On other hand, chronic inflammation and a decrease in anabolic hormones, such as insulin-like growth factor-1 (IGF-1), essential for muscle repair and growth [10,11] further exacerbate muscle tissue alterations, consequently diminishing the capacity for force production [12]. In this context, Maktouf et al. [8] showed that older adults with obesity possess lower relative maximal plantar flexor strength. However, it remains unknown whether obesity in older adults exacerbates the decline in RFD. This is particularly pertinent, as older adults with obesity are at higher risk of falling than younger adults [13].

Walking is an indicator of autonomy [14] and plays a key role in disease prevention and weight management in older adults [15]. It also serves as a crucial diagnostic criterion for conditions like sarcopenia and frailty [16]. Recent research has indicated that obese older adults face a 1.5 to 5 times higher risk of developing walking limitations compared to their normal-weight peers [13]. In this context, Laroche et al. [17] showed that older adults with obesity spend less time in the single support phase of walking and more time in the double support phase than older adults of normal weight. During the propulsion phase, the role of plantar flexors is pivotal for propelling the body forward and facilitating step transition [18], underscoring their notable association with walking speed [19]. In this context, Maktouf et al. [20] demonstrated that gait parameter alterations in older adults with obesity are attributed to increased activation of the PF, with body mass contributing 87% of the variation in this heightened activation. However, there is a notable gap in the literature concerning the relationship between the RFD of PF and walking speed, especially among older adults with obesity. Addressing this research gap is essential for developing targeted interventions and ensuring that therapeutic strategies are based on a thorough understanding of the underlying dynamics.

The objectives of this study were as follows: (i) to investigate the influence of obesity on RFD in older adults with sarcopenia, and (ii) to examine the relationship between neuromuscular markers and gait speed in older adults with obesity (SO).

## 2. Materials and Methods

### 2.1. Study Design

**Figure 1:**
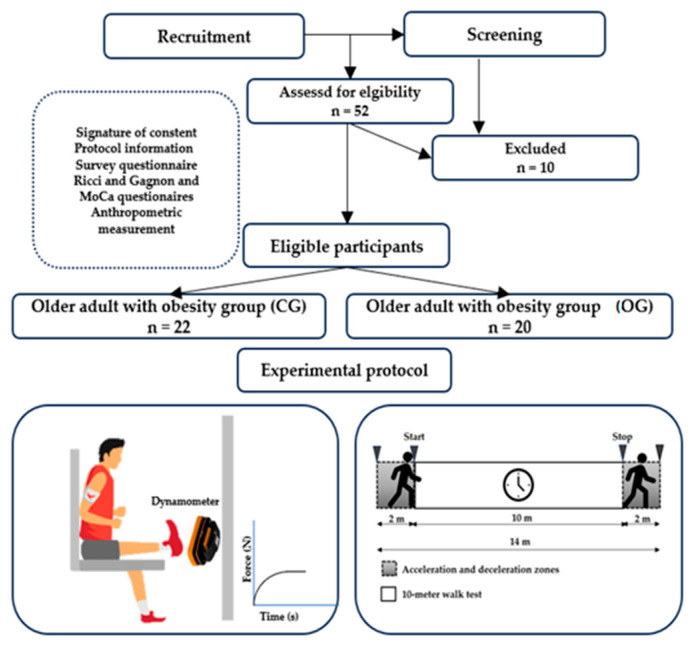
Study design.

The study was designed following an analytical cross-sectional approach, as depicted in Figure 1. The process involved a 4-week recruitment phase, a 3-week screening phase, and culminated in a 2 h evaluation session that included health questionnaires, anthropometric measurements, a 10 m walking test, and neuromuscular assessments. 

### 2.2. Recruitment 

Participants were recruited from various care centers through announcements and medical staff compiled lists of volunteers for researchers. Eligibility for older adults required being over 65 years old. Based on body mass index (BMI) criteria, those with a BMI under 25 kg/m^2^ were placed in the control group (CG), and individuals with a BMI over 30 kg/m^2^ were categorized into the older adults with obesity group (OG). Exclusion criteria included neurological or cognitive impairments, severe cardiovascular diseases, significant lower limb musculoskeletal problems, other major comorbidities or chronic diseases, use of medication affecting test outcomes, or a Montreal Cognitive Assessment (MoCA) score below 26.

### 2.3. Experimental Protocol

All evaluations were conducted in a designated clinical examination room under consistent environmental conditions, overseen by one trained assessor. Participants were provided with a standardized set of verbal instructions before the assessments to ensure familiarity with the procedures. 

#### 2.3.1. Gait Speed Evaluation

Gait speed was assessed using a chronometer during the 10 m walk test (10MWT), as outlined in Figure 1. Participants were instructed to walk along a 14 m corridor, which included a 2 m acceleration zone, a 10 m measurement zone for the 10MWT, and a 2 m deceleration zone [21]. To ensure accuracy, only the time taken to cover the distance between the 3rd and 13th meters was recorded, effectively eliminating the influence of the acceleration and deceleration phases. The timing commenced as soon as the participant’s toes crossed the 2nd meter mark and stopped when they crossed the 12th meter mark, in accordance with the American Physical Therapy Association Clinical Practice Guidelines [22]. Each participant performed three trials of the 10MWT, with a minimum rest period of 30 s between each trial. 

For data processing, the average time from the three trials was used to calculate the 10MWT speed (m/s) as follows:

Gait speed (m/s) = time (s)/distance (m), where distance = 10 m

#### 2.3.2. Neuromuscular Parameters Evaluation

Neuromuscular parameters of the PF of the dominant leg were measured using a dynamometer (K-Force, Kinvent, Montpellier, France) with sampling rate of 1000 Hz and accuracy of 100 g. Participants were instructed to maintain contact between their back, buttocks, and thigh with the chair while keeping their leg stretched horizontally [8].

The protocol commenced with a dynamic plantar flexion of the ankle as a warm-up. Then, participants performed two explosive maximal isometric voluntary contractions (MVCs), each lasting approximately 1 s, interspersed with 20 s rest periods. The absolute MVC from these two trials was recorded (Fmax, N). To calculate the relative force (R-Fmax, N/kg), the A-Fmax was normalized to the participant’s body mass (A-Fmax/Body mass, N/kg).

**Figure 2:**
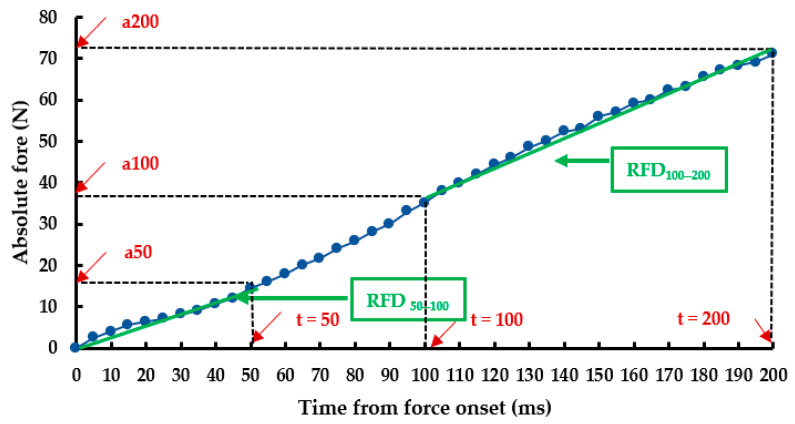
Force–time curve from force onset to 200 ms during maximal voluntary contraction of plantar flexors.

**Figure 3:**
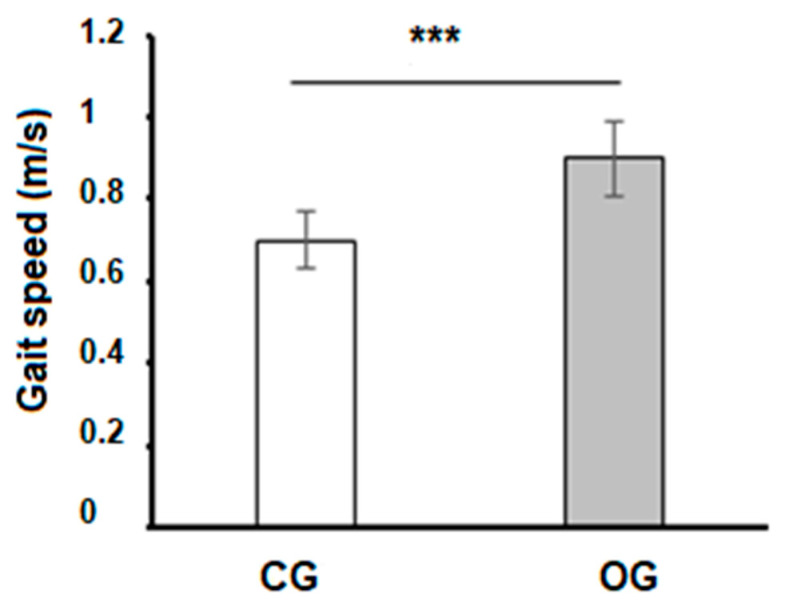
Comparison of gait speed between groups. OG—older adults with obesity; CG—control group; *** *p* < 0.001.

For data processing, the numeric force signal from the dynamometer was filtered using a second-order zero-lag Butterworth low-pass filter with a 40-Hz cutoff frequency, employing Matlab (The MathWorks Inc., Natick, MA, USA) [23]. The onset of each contraction was identified using a second derivative method. The force signal analysis commenced from the onset for a 200 ms time window (Figure 2). Absolute force values were extracted at 50 ms (F50), 100 ms (F100), and 200 ms (F200) intervals. These values were normalized to body mass (R–F50, R–F100, and R–F200) and to Fmax (50 Fmax, 100 Fmax, and 200 Fmax (%)). The RFD–time curve was then calculated through the first derivative of the force signal (Δ force/Δ time) at each overlapping interval within the 200 ms continuum (Figure 3) [23]. This curve was also low-pass filtered at a 50 Hz cutoff frequency. The early RFD, from onset to 50 ms (RFD_0–50_), and the late RFD, from 100 to 200 ms (RFD_100–200),_ were extracted and normalized to body mass (N·kg^−1^·s^−1^)

### 2.4. Statistical Analysis

The sample size was calculated using the freeware G*Power (version 3.1.9.4) [24]. The *t*-test was predefined for power analysis. The estimation was based on predefined controls for type I errors (alpha = 0.05) and Type II errors (beta = 0.60), with a moderate level of estimated effect size (r = 0.30) [15]. Under these settings, 38 participants were required for the minimum sample size.

For statistical analysis, Jamovi software (version 2.3, Sydney, Australia) was utilized [25]. The Shapiro–Wilk test and the Levene test were employed to ascertain data normality and variance homogeneity, respectively. Upon confirming that the data adhered to these assumptions, an independent samples *t*-test was conducted to identify the variance between the groups. Moreover, a Pearson correlation analysis was performed to pinpoint the parameters strongly associated with gait speed in the two groups.

## 3. Results

### 3.1. Participants

An initial group of 52 older adults was recruited. Following a thorough application of inclusion and exclusion criteria, 45 participants met the study’s eligibility requirements. Regrettably, three individuals could not complete the study, resulting in a final cohort of 42 participants who completed the study in its entirety.

Statistical analysis showed no significant differences in mean age, height and LBM between the OG and CG. Nonetheless, BM (*p* < 0.001), BMI (*p* < 0.001), body fat (*p* < 0.001) and FBM (*p* < 0.001) were notably higher in the OG compared to the CG (Table 1).

### 3.2. Gait Speed

The average gait speed was lower in the OG compared to the CG (*p* < 0.001) (Figure 3).

### 3.3. Neuromuscular Parameters

The neuromuscular parameters during the MVC test are detailed in Table 2. There was no significant difference between the OG and CG in terms of Fmax. However, F50 (*p* < 0.001), F100 (*p* = 0.015), and F200 (*p* < 0.001) were lower in the OG. When normalized to BM, R-F50 (*p* < 0.001; d = −1.80), R-F100 (*p* < 0.001; d = −2.89), and R-F200 (*p* < 0.001; d = −3.13) were also higher in the CG. In addition, RFD_50/100_ and RFD_100/200_ were lower in the OG compared to the CG (*p* < 0.001 and *p* < 0.001, respectively).

### 3.4. Relationships between Neuromusclar Parameters and Gait Speed

**Figure 4:**
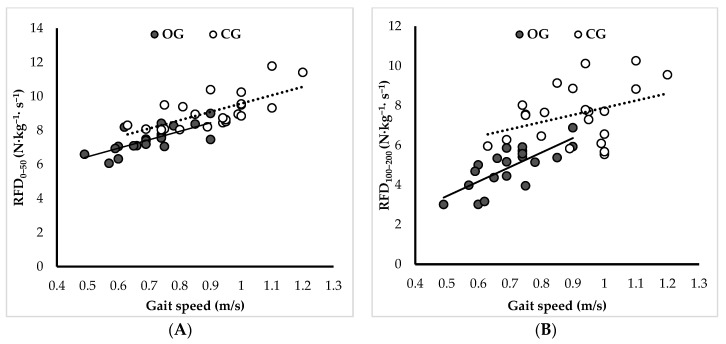
(**A**) Relationship between RFD_0–50_ and gait speed. (**B**) Relationship between RFD_100–200_ and gait speed.

Gait speed exhibited significant correlations with neuromuscular parameters within the CG and OG, as detailed in Table 3. Specifically, within the OG, the most pronounced correlation was identified with RFD_0/50_ (r = 0.69, *p* < 0.001). Additionally, the correlation with RFD_100/200_ was also notably high (r = 0.66, *p* < 0.001) (Figure 4). Conversely, for the CG, the highest correlation was noted with RFD_100/200_ (r = 0.56, *p* < 0.001) followed by RFD_0/50_ (r = 0.52, *p* < 0.01) (Figure 4). Moreover, the gait speeds of the OG and CG were positively correlated with R-Fmax (r = 0.42, *p* < 0.05, and r = 0.39, *p* < 0.05, respectively). However, absolute Fmax exhibited no significant correlations with gait speed within either the CG or the OG.

## 4. Discussion

The primary aim of this study was to discern the impact of obesity on the neuromuscular parameters of plantar flexors in older adults. The findings underscored the significant influence of obesity on neuromuscular parameters, particularly the RFD. Our secondary objective concerned the relationship between these neuromuscular parameters and gait speed in older adults with obesity. Notably, the RFD of plantar flexors stood out as the predominant factor affecting gait speed, notably surpassing the impact of the relative maximal force of plantar flexors.

Our study found that obesity had no significant impact on the absolute maximal force generated by the plantar flexors, a finding consistent with the results of Maktouf et al. [8], who also observed no differences between older adults with normal weight and their obese counterparts. However, when forces were normalized to body mass, both the maximal and submaximal forces of plantar flexors were notably lower in the OG. This is in alignment with multiple studies examining the effects of obesity on muscle strength in young adults [26,27]. This phenomenon may be partially explained by the observations of Tomlinson et al. [11], who noted that the most significant impact of combined aging and adiposity was on the rate of muscle volume loss. One plausible mechanism underlying this accelerated muscle loss is that obesity exacerbates the challenges posed by sarcopenia [12]. It does so by exerting additional mechanical stress on the musculoskeletal system, particularly due to the need to support elevated adipose tissue weight [28]. Beyond serving merely as an energy store, adipose tissue is a dynamic endocrine organ that secretes an array of hormones and pro-inflammatory cytokines, thereby amplifying biochemical stress in the body [29]. Chronic adiposity results in elevated levels of circulating pro-inflammatory cytokines such as TNF-α, IL-1α, IL-6, and CRP, which contribute to both acute and chronic systemic inflammation [30]. These inflammatory agents negatively impact skeletal muscle by promoting protein degradation over synthesis, ultimately leading to muscle wasting or atrophy [10]. Further complicating the scenario is the association between obesity and declines in anabolic hormones, specifically insulin-like growth factor-1 (IGF-1), which plays a crucial role in muscle repair and growth [31].

The innovative aspect of this study was the assessment of the RFD, identified as a crucial parameter for evaluating mobility in vulnerable populations. Our findings reveal a marked influence of obesity on RFD parameters. Specifically, the values for RFD_0/50_ and RFD_100/200_ decreased by 18.4% and 35.2%, respectively, in the OG when compared to the CG. Expanding upon related findings, Olmos et al. [1] distinctly highlighted that in older adults without obesity the deterioration in the late RFD (i.e., RFD_100/200_) is markedly more pronounced, demonstrating a significant reduction when compared with the early RFD. This finding reveals the heightened sensitivity of late-stage neuromuscular functionality to age-related declines, particularly outside the context of obesity. Drawing on these insights, we suggest that the detrimental effects on early rapid force are likely to be obesity-specific in older adults, while the impact on late rapid force seems to exacerbate existing sarcopenia-related impairments. This hypothesis gains credence from the robust correlation identified within the CG, where the relationship between the late RFD and gait speed was found to be more profound than with the early RFD (Figure 4).

The impact of obesity on both early and late rapid force in older adults can be elucidated by examining multiple factors, such as motor unit recruitment [32], intrinsic muscle properties [11,33], and systemic inflammation [10,12]. Early rapid force is predominantly associated with initial motor unit recruitment and firing rates, as well as intrinsic muscle attributes such as fiber type composition and calcium kinetics [6]. Obesity might specifically affect the early RFD due to the increased mechanical stress it places on the musculoskeletal system, thereby affecting the efficiency of motor unit recruitment and the contractile properties of muscle fibers. Moreover, adipose tissue in older adults with obesity functions as a dynamic endocrine organ, secreting an array of hormones and pro-inflammatory cytokines, like TNF-a, IL-1a, IL-6, and CRP [10]. This heightened state of systemic inflammation could adversely affect the early RFD by causing protein degradation to outpace synthesis, leading to muscle atrophy and reduced contractile capabilities. On the other hand, late rapid force measures are more strongly influenced by factors such as maximal strength, muscle size, tendon stiffness, and pennation angle [7]. Obesity can exacerbate the loss of muscle mass—often referred to as sarcopenia—which is already compromised in older adults. The combined effect of obesity and aging leads to a decline in maximal strength and muscle size, which, in turn, significantly impacts the late RFD. The accelerated muscle loss may be further exacerbated by chronic inflammation and a decline in anabolic hormones, such as insulin-like growth factor-1 (IGF-1), which are crucial for muscle repair and growth [10,11].

Our research presents compelling evidence underscoring the RFD as a crucial determinant of gait speed among older adults, irrespective of obesity status, although the effects observed in each group suggest potentially distinct mechanisms influencing early and late phases of the RFD. These findings align with previous research on frail populations, which identified the RFD as a critical predictor of various physical functionalities [33,34]. Such functionalities encompass a range of activities, from standing up from a seated position to performing timed up-and-go tests and achieving both casual and maximal walking speed among older adults [4]. Furthermore, our results resonate with other studies highlighting the paramount importance of muscle power over mere muscle strength in influencing walking speed and, consequently, the risk of falls [1,4].

A critical question that warrants discussion is why the RFD is a more salient predictor of gait speed than maximal force. One plausible explanation is that impairments of quick force generation could constrain an individual’s capacity to engage in rapid movements, especially in activities requiring sequential agonist and antagonist muscle contractions, such as walking [3]. Many daily activities necessitate the rapid application of force over a short duration (i.e., RFD capacity). For instance, a noticeable surge in force within approximately 200 milliseconds is essential when an older adult stands up from a seated position [1]. Furthermore, the ability to avert a fall is not solely reliant on the production of maximal force but is also contingent on the speed of motor responses [35]. This underscores the functional significance of the rate at which submaximal force can be generated (i.e., RFD). Hence, the RFD serves a dual role; it is not only a performance determinant in functional tasks that demand more power than force but is also a pivotal metric for assessing fall risk [3,4].

### 4.1. Limitations and Perspectives

Our study has several limitations that warrant attention. First and foremost, the small cohort size restricts the generalizability of our findings, particularly given the heterogeneity often observed in older adults with obesity. These populations can sometimes present a complex interplay of factors, resulting in characteristics that may not be wholly reflective of those in our study sample. Crucially, the analysis did not account for the potential for multi-collinearity between the features under consideration. While correlations provide valuable insights, they do not discern between the complex interdependencies that might exist between multiple variables, which could obscure the true associations and predictive capacities within the model. Another key limitation lies in the non-utilization of electromyography, which could have provided further insights into the neurological mechanisms involved in force development and muscle function. The exclusion of this analytical tool leaves certain questions unanswered and calls for additional research to elucidate these mechanisms more comprehensively. These limitations underscore the necessity for further research, including studies with larger and more diverse samples, the inclusion of electromyography, and sophisticated statistical modeling that addresses multi-collinearity. Such research would furnish a more comprehensive understanding of how obesity interacts with neuromuscular capacities and mobility.

### 4.2. Practical Recommendations

Considering our study’s findings, which emphasize the critical role of the RFD in physical function, clinicians are urged to integrate RFD measurements into their standard neuromuscular assessments for a more nuanced understanding, particularly in vulnerable populations like older adults with obesity. Based on this comprehensive evaluation, tailored intervention programs should be designed to improve the RFD and thereby enhance essential functional capacities, such as walking speed. Instead of solely focusing on maximal force, exercise regimens should prioritize muscle power, incorporating high-intensity, explosive movements that mimic real-world scenarios requiring rapid force generation. This approach is particularly pertinent for fall prevention programs, as the RFD is a significant predictor of fall risks.

## 5. Conclusions

Our findings reveal that obesity has a pronounced negative impact on RFD. Specifically, the adverse effects on the early RFD appear to be obesity-specific in older adults with obesity, while also exacerbating the existing impairments related to sarcopenia in the late RFD. Significantly, the early RFD emerges as the dominant factor influencing gait speed, far surpassing the impact of the relative maximal force of plantar flexors. This underscores the critical diagnostic and prognostic role that the RFD could play in the management of this vulnerable population. Furthermore, rather than concentrating exclusively on improving maximal force, physical exercise regimens should prioritize boosting muscle power to better address the nuanced challenges faced by older adults with obesity.

## Figures and Tables

**Table 1:** Anthropometric characteristics of groups.

Parameters	Groups	Mean ± SD
Age (years)	OG	77.7 ± 2.9
	CG	81.1 ± 4.0
Height (cm)	OG	162.9 ± 6.3
	CG	166.0 ± 7.5
Body mass (kg)	OG	91.0 ± 3.9 ***
	CG	68.74 ± 5.5
Body mass index (kg/h^2^)	OG	34.5 ± 3.2 ***
	CG	25.1 ± 3.4
Body fat (%)	OG	35.0 ± 6.34 ***
	CG	17.7 ± 1.98
Fat body mass (kg)	OG	32.0 ± 6.9 ***
	CG	12.2 ± 1.5
Lean body mass (kg)	OG	58.9 ± 4.1 ***
	CG	56.6 ± 5.0

OG—older adults with obesity; CG—control group; *** *p* < 0.001.

**Table 2:** Neuromuscular parameters of plantar flexors.

Parameters	Groups	Mean ± SD
Fmax (N)	OG	192.69 ± 15.41
	CG	194.89 ± 13.28
R-Fmax (N/kg)	OG	2.12 ± 0.22 ***
	CG	2.86 ± 0.36
F50 (N)	OG	31.18 ± 2.12 ***
	CG	33.80 ± 2.65
R-F50 (N/kg)	OG	0.37 ± 0.03 ***
	CG	0.45 ± 0.05
F100 (N)	OG	69.53 ± 5.45 *
	CG	73.51 ± 4.73
R–F100 (N/kg)	OG	0.76 ± 0.08 ***
	CG	1.07 ± 0.12
F200 (N)	OG	113.96 ± 8.93 ***
	CG	124.73 ± 8.48
R–F200 (N/kg)	OG	1.25 ± 0.13
	CG	1.82 ± 0.21
50–Fmax (%)	OG	17.60 ± 1.56
	CG	16.02 ± 0.96
100–Fmax (%)	OG	36.14 ± 2.01
	CG	37.81 ± 2.57
200–Fmax (%)	OG	59.40 ± 5.64 *
	CG	64.16 ± 4.72
RFD_0–50_ (N·kg^−1^·s^−1^)	OG	7.45 ± 0.74 ***
	CG	9.13 ± 1.04
RFD_100–200_ (N·kg^−1^·s^−1^)	OG	4.90 ± 1.06 ***
	CG	7.57 ± 1.43

OG—older adults with obesity; CG—control group; RFD—rate of force development; F50, F100 and F200—absolute force at 50, 100 and 200 ms; R–F50, R–F100, R–F200—relative force at 50, 100 and 200 ms; 50 Fmax, 100 Fmax, 200 Fmax—percentage force achieved at 50, 100 and 200 ms relative to the absolute Fmax; Fmax—absolute maximal force; R-Fmax—relative maximal force; SD—standard deviation; * *p* < 0.05, *** *p* < 0.001.

**Table 3:** Correlation analysis between neuromuscular parameters and gait speed.

Neuromuscular Parameters	Group	Gait Speed (m/s)	
		r	*p*
RFD_0/50_ (N·kg^−1^·s^−1^)	OG	0.69	<0.001
	CG	0.52	<0.01
RFD_100/200_ (N·kg^−1^·s^−1^)	OG	0.66	<0.001
	CG	0.56	<0.001
Fmax (N)	OG	0.23	NS
	CG	0.28	NS
R–Fmax (N/kg)	OG	0.42	<0.05
	CG	0.39	<0.05
50–Fmax (%)	OG	0.27	NS
	CG	0.33	NS
100–Fmax (%)	OG	0.20	NS
	CG	0.45	<0.05
200–Fmax (%)	OG	0.05	NS
	CG	0.49	<0.05

CG—control group; OG—obese group; RFD—rate of force development; 50 Fmax, 100 Fmax, 200 Fmax: percentage force achieved at 50, 100 and 200 ms relative to the absolute Fmax; Fmax—absolute maximal force; R–Fmax—relative maximal force; NS—no significant difference.

## Data Availability

The data presented in this study are available on request from the corresponding author.

## References

[1] Olmos A.A., Stratton M.T., Ha P.L., Dalton B.E., VanDusseldorp T.A., Mangine G.T., Feito Y., Poisal M.J., Jones J.A., Smith T.M. (2020). Early and Late Rapid Torque Characteristics and Select Physiological Correlates in Middle-Aged and Older Males. PLoS ONE.

[2] Aagaard P., Simonsen E.B., Andersen J.L., Magnusson P., Dyhre-Poulsen P. (2002). Neural Adaptation to Resistance Training: Changes in Evoked V-Wave and H-Reflex Responses. J. Appl. Physiol..

[3] Gerstner G.R., Thompson B.J., Rosenberg J.G., Sobolewski E.J., Scharville M.J., Ryan E.D. (2017). Neural and Muscular Contributions to the Age-Related Reductions in Rapid Strength. Med. Sci. Sports Exerc..

[4] Hester G.M., Ha P.L., Dalton B.E., Vandusseldorp T.A., Olmos A.A., Stratton M.T., Bailly A.R., Vroman T.M. (2021). Rate of Force Development as a Predictor of Mobility in Community-Dwelling Older Adults. J. Geriatr. Phys. Ther..

[5] Thompson B.J., Ryan E.D., Herda T.J., Costa P.B., Herda A.A., Cramer J.T. (2014). Age-Related Changes in the Rate of Muscle Activation and Rapid Force Characteristics. Age.

[6] Klass M., Baudry S., Duchateau J. (2008). Age-Related Decline in Rate of Torque Development Is Accompanied by Lower Maximal Motor Unit Discharge Frequency during Fast Contractions. J. Appl. Physiol..

[7] Andersen L.L., Aagaard P. (2006). Influence of Maximal Muscle Strength and Intrinsic Muscle Contractile Properties on Contractile Rate of Force Development. Eur. J. Appl. Physiol..

[8] Maktouf W., Durand S., Boyas S., Pouliquen C., Beaune B. (2018). Combined Effects of Aging and Obesity on Postural Control, Muscle Activity and Maximal Voluntary Force of Muscles Mobilizing Ankle Joint. J. Biomech..

[9] Hilton T.N., Tuttle L.J., Bohnert K.L., Mueller M.J., Sinacore D.R. (2008). Excessive Adipose Tissue Infiltration in Skeletal Muscle in Individuals with Obesity, Diabetes Mellitus, and Peripheral Neuropathy: Association with Performance and Function. Phys. Ther..

[10] Erskine R.M., Tomlinson D.J., Morse C.I., Winwood K., Hampson P., Lord J.M., Onambélé G.L. (2017). The Individual and Combined Effects of Obesity- and Ageing-Induced Systemic Inflammation on Human Skeletal Muscle Properties. Int. J. Obes..

[11] Tomlinson D.J., Erskine R.M., Morse C.I., Winwood K., Onambélé-Pearson G.L. (2014). Combined Effects of Body Composition and Ageing on Joint Torque, Muscle Activation and Co-Contraction in Sedentary Women. Age.

[12] Tomlinson D.J., Erskine R.M., Morse C.I., Winwood K., Onambélé-Pearson G. (2016). The Impact of Obesity on Skeletal Muscle Strength and Structure through Adolescence to Old Age. Biogerontology.

[13] Handrigan G.A., Maltais N., Gagné M., Lamontagne P., Hamel D., Teasdale N., Hue O., Corbeil P., Brown J.P., Jean S. (2017). Sex-Specific Association between Obesity and Self-Reported Falls and Injuries among Community-Dwelling Canadians Aged 65 Years and Older. Osteoporos. Int..

[14] Xu D., Zhou H., Quan W., Jiang X., Liang M., Li S., Ugbolue U.C., Baker J.S., Gusztav F., Ma X. (2023). A New Method Proposed for Realizing Human Gait Pattern Recognition: Inspirations for the Application of Sports and Clinical Gait Analysis. Gait Posture.

[15] Ferhi H., Gaied-Chortane S., Durand S., Beaune B., Boyas S., Maktouf W. (2023). Effects of Physical Activity Program on Body Composition, Physical Performance, and Neuromuscular Strategies during Walking in Older Adults with Sarcopenic Obesity: Randomized Controlled Trial. Healthcare.

[16] Dommershuijsen L.J., Ragunathan J., Ruiter T.R., Groothof D., Mattace-Raso F.U.S., Ikram M.A., Polinder-Bos H.A. (2022). Gait Speed Reference Values in Community-Dwelling Older Adults—Cross-Sectional Analysis from the Rotterdam Study. Exp. Gerontol..

[17] Laroche D.P., Marques N.R., Shumila H.N., Logan C.R., Laurent R.S., Goncąlves M. (2015). Excess Body Weight and Gait Influence Energy Cost of Walking in Older Adults. Med. Sci. Sports Exerc..

[18] Ellis R.G., Sumner B.J., Kram R. (2014). Muscle Contributions to Propulsion and Braking during Walking and Running: Insight from External Force Perturbations. Gait Posture.

[19] Tavakkoli Oskouei S., Malliaras P., Jansons P., Hill K., Soh S.E., Jaberzadeh S., Perraton L. (2021). Is Ankle Plantar Flexor Strength Associated with Balance and Walking Speed in Healthy People? A Systematic Review and Meta-Analysis. Phys. Ther..

[20] Maktouf W., Durand S., Boyas S., Pouliquen C., Beaune B. (2020). Interactions among Obesity and Age-Related Effects on the Gait Pattern and Muscle Activity across the Ankle Joint. Exp. Gerontol..

[21] Cleland B.T., Alex T., Madhavan S. (2023). Concurrent Validity of Walking Speed Measured by a Wearable Sensor and a Stopwatch during the 10-Meter Walk Test in Individuals with Stroke. Gait Posture.

[22] Moore J.L., Potter K., Blankshain K., Kaplan S.L., O’Dwyer L.C., Sullivan J.E. (2018). A Core Set of Outcome Measures for Adults with Neurologic Conditions Undergoing Rehabilitation. J. Neurol. Phys. Ther..

[23] Chatrenet A., Piccoli G., Audebrand J.M., Torreggiani M., Barbieux J., Vaillant C., Morel B., Durand S., Beaune B. (2023). Analysis of the Rate of Force Development Reveals High Neuromuscular Fatigability in Elderly Patients with Chronic Kidney Disease. J. Cachexia Sarcopenia Muscle.

[24] Faul F., Erdfelder E., Lang A.G., Buchner A. (2007). G*Power 3: A Flexible Statistical Power Analysis Program for the Social, Behavioral, and Biomedical Sciences. Proc. Behav. Res. Methods.

[25] Soumboundou S., Ndiaye M.L., Marcellin Nouaman N., Farhat O., Abdou Lecor P. (2023). Three-Dimensional Anthropometric Study of the Facial Morphology of Black African Senegalese: 3D Photogrammetric Approach. J. Oral. Biol. Craniofac. Res..

[26] Lafortuna C.L., Maffiuletti N.A., Agosti F., Sartorio A. (2005). Gender Variations of Body Composition, Muscle Strength and Power Output in Morbid Obesity. Int. J. Obes..

[27] Abdelmoula A., Martin V., Bouchant A., Walrand S., Lavet C., Taillardat M., Maffiuletti N.A., Boisseau N., Duché P., Ratel S. (2012). Knee Extension Strength in Obese and Nonobese Male Adolescents. Appl. Physiol. Nutr. Metab..

[28] Maffiuletti N.A., Jubeau M., Munzinger U., Bizzini M., Agosti F., De Col A., Lafortuna C.L., Sartorio A. (2007). Differences in Quadriceps Muscle Strength and Fatigue between Lean and Obese Subjects. Eur. J. Appl. Physiol..

[29] Hotamisligil G.S., Arner P., Caro J.F., Atkinson R.L., Spiegelman B.M. (1995). Increased Adipose Tissue Expression of Tumor Necrosis Factor-Alpha in Human Obesity and Insulin Resistance. J. Clin. Investig..

[30] Park H.S., Park J.Y., Yu R. (2005). Relationship of Obesity and Visceral Adiposity with Serum Concentrations of CRP, TNF-α and IL-6. Diabetes Res. Clin. Pract..

[31] Galli G., Pinchera A., Piaggi P., Fierabracci P., Giannetti M., Querci G., Scartabelli G., Manetti L., Ceccarini G., Martinelli S. (2012). Serum Insulin-like Growth Factor-1 Concentrations Are Reduced in Severely Obese Women and Raise after Weight Loss Induced by Laparoscopic Adjustable Gastric Banding. Obes. Surg..

[32] Hammond K.G., Pfeiffer R.F., LeDoux M.S., Schilling B.K. (2017). Neuromuscular Rate of Force Development Deficit in Parkinson Disease. Clin. Biomech..

[33] Maffiuletti N.A., Aagaard P., Blazevich A.J., Folland J., Tillin N., Duchateau J. (2016). Rate of Force Development: Physiological and Methodological Considerations. Eur. J. Appl. Physiol..

[34] Mathern R.M., Anhorn M., Uygur M. (2019). A Novel Method to Assess Rate of Force Relaxation: Reliability and Comparisons with Rate of Force Development across Various Muscles. Eur. J. Appl. Physiol..

[35] Bellumori M., Jaric S., Knight C.A. (2013). Age-Related Decline in the Rate of Force Development Scaling Factor. Mot. Control.

